# A Functional Genomic Yeast Screen to Identify Pathogenic Bacterial Proteins 

**DOI:** 10.1371/journal.ppat.0040009

**Published:** 2008-01-18

**Authors:** Naomi L Slagowski, Roger W Kramer, Monica F Morrison, Joshua LaBaer, Cammie F Lesser

**Affiliations:** 1 Department of Medicine, Division of Infectious Diseases, Massachusetts General Hospital, Harvard Medical School, Cambridge, Massachusetts, United States of America; 2 Harvard Institute of Proteomics, Harvard Medical School, Cambridge, Massachusetts, United States of America; Tufts University School of Medicine, United States of America

## Abstract

Many bacterial pathogens promote infection and cause disease by directly injecting into host cells proteins that manipulate eukaryotic cellular processes. Identification of these translocated proteins is essential to understanding pathogenesis. Yet, their identification remains limited. This, in part, is due to their general sequence uniqueness, which confounds homology-based identification by comparative genomic methods. In addition, their absence often does not result in phenotypes in virulence assays limiting functional genetic screens. Translocated proteins have been observed to confer toxic phenotypes when expressed in the yeast Saccharomyces cerevisiae. This observation suggests that yeast growth inhibition can be used as an indicator of protein translocation in functional genomic screens. However, limited information is available regarding the behavior of non-translocated proteins in yeast. We developed a semi-automated quantitative assay to monitor the growth of hundreds of yeast strains in parallel. We observed that expression of half of the 19 *Shigella* translocated proteins tested but almost none of the 20 non-translocated *Shigella* proteins nor ∼1,000 Francisella tularensis proteins significantly inhibited yeast growth. Not only does this study establish that yeast growth inhibition is a sensitive and specific indicator of translocated proteins, but we also identified a new substrate of the *Shigella* type III secretion system (TTSS), IpaJ, previously missed by other experimental approaches. In those cases where the mechanisms of action of the translocated proteins are known, significant yeast growth inhibition correlated with the targeting of conserved cellular processes. By providing positive rather than negative indication of activity our assay complements existing approaches for identification of translocated proteins. In addition, because this assay only requires genomic DNA it is particularly valuable for studying pathogens that are difficult to genetically manipulate or dangerous to culture.

## Author Summary

Many bacterial pathogens promote infection and ultimately cause disease, in part, through the actions of proteins that the bacteria directly inject into host cells. These proteins subvert host cell processes to favor survival of the pathogen. The identification of such proteins can be limited since many of the injected proteins lack homology with other virulence proteins and pathogens that no longer express the proteins are often unimpaired in conventional assays of pathogenesis. Many of these proteins target cellular processes conserved from mammals to yeast, and overexpression of these proteins in yeast results in growth inhibition. We have established a high throughput growth assay amenable to systematically screening open reading frames from bacterial pathogens for those that inhibit yeast growth. We observe that yeast growth inhibition is a sensitive and specific indicator of proteins that are injected into host cells. Expression of about half of the injected bacterial proteins but almost none of the bacteria-confined proteins results in yeast growth inhibition. Since this assay only requires genomic DNA it is particularly valuable for studying pathogens that are difficult to genetically manipulate or dangerous to grow in the laboratory.

## Introduction

The complete genomes of over 150 bacteria including many pathogens have been sequenced. Comparative genomics can predict functions for up to 75% of the annotated open reading frames (ORFs), the majority of which encode housekeeping proteins. Nevertheless, the functions of hundreds of proteins typically defy prediction. Included within this set are bacterial proteins that play a role in the development of infections. For example, both intra- and extra-cellular bacterial pathogens, like their viral counterparts, affect intracellular processes by directly delivering proteins into the cytoplasm of host cells by processes such as type III, type IV and type VI secretion [1–3]. The components of the complex protein “machines” that mediate translocation are relatively well conserved, but their substrates, the translocated proteins, are poorly conserved and rarely share detectable translocation signal sequences.

Numerous genome-wide experimental approaches have been undertaken to identify bacterial proteins involved in pathogenesis. These include screening banks of transposon-generated mutants for alterations in pathogenesis [4] as well as identifying proteins selectively expressed during infections [5]. Although these screens are effective they are limited to bacteria that are genetically manipulable and have susceptible animal or cell culture models. In addition, since translocated bacterial proteins are often functionally redundant they are often missed in genetic screens for mutants defective in specific steps of pathogenesis [6]. Alternative complementary experimental approaches are needed to facilitate rapid screening of genomes to identify potential pathogenic proteins on which to focus subsequent studies.

Although yeast cannot serve as a physiologic model of human infection, S. cerevisiae is an established organism for characterizing translocated bacterial proteins that target conserved eukaryotic physiology. For example, translocated proteins that alter the mammalian actin cytoskeleton as well as the secretory and signal transduction pathways have been demonstrated to target analogous processes in yeast [7–13]. Perhaps unsurprisingly, such targeting is often detrimental to yeast and results in measurable growth inhibition. This suggests that yeast growth inhibition is a sensitive indicator of translocated proteins. Indeed, expression of three of the five known Yersinia pseudotuberculosis effector proteins and four of the eight LEE (locus of enterocyte effacement) encoded enteropathogenic Escherichia coli effector proteins inhibit yeast growth [7,14]. Of note, in each of these cases, the effector proteins were expressed at relatively high levels in yeast as fusion proteins.

There is limited information available on the specificity of yeast growth inhibition as an indicator of translocated proteins. A recent genome-wide screen for Legionella pneumophilia proteins that inhibit yeast growth suggests that growth inhibition due to expression of non-translocated proteins is rare. In this screen that covered ∼60% of the *Legionella* proteome, fragments of only nine *Legionella* proteins, three translocated and six non-translocated proteins, inhibited yeast growth [8]. However, this study was likely limited because each *Legionella* protein fragment was fused to the classic SV40 nuclear localization signal, a hemagglutinin epitope tag and the acidic transcription activation domain of B42. Since fusion to the SV40 NLS is often sufficient to direct nuclear localization of normally cytoplasmic proteins, altered targeting could interfere with the activity of *Legionella* proteins. Similarly, the acidic activation domain could disrupt function. Furthermore, the *Legionella* proteins were expressed at relatively high levels in yeast.

The observations summarized above suggested that yeast growth inhibition could be used as a functional genomic screen to identify previously unknown virulence proteins that target host cells. In order to determine if this is indeed the case as well as to optimize such screens, we systematically characterized the behavior in yeast of known translocated and non-translocated bacterial proteins when expressed at either relatively high or low-levels on their own or fused to GFP. We developed an economical high throughput growth assay to facilitate such studies on a genome-wide scale. We observed that expression of half of the 19 *Shigella* translocated proteins tested but almost none of the 20 non-translocated *Shigella* proteins nor >1,000 F. tularensis proteins significantly inhibit yeast growth. Not only does this study establish that yeast growth inhibition is a sensitive and specific indicator of translocated proteins, but we also identified a new substrate of the *Shigella* type III secretion system (TTSS), IpaJ, previously missed by other experimental approaches. These observations demonstrate that yeast growth inhibition can be used as a functional genomic screen to identify pathogenic proteins.

## Results/Discussion

### Selection of *Shigella* Proteins from Virulence Plasmid for Yeast Growth Assays

*Shigella,* the causative agent of bacterial dysentery, is an intracellular pathogen that mediates its own uptake and subsequent dissemination between host cells in part by the action of translocated effector proteins (for review [15]). All pathogenic *Shigella* species contain a virulence plasmid that encodes ∼106 full-length ORFs including one of the largest known sets of proteins translocated by a TTSS [16,17]. This plasmid encodes at least 14 known substrates of the TTSS [18–23] as well as seven additional proteins have been proposed to be TTSS substrates based on homology [16]. A third of these ORFs are organized within two operons required for entry of *Shigella* into host cells. These operons encode components and regulators of a TTSS as well as a few translocated proteins [16,17]. The GC-content of all 34 genes present in these two operons is ∼34% while the GC-content of *Shigella* chromosomal genes is generally >50%. Twenty-two additional low GC-content (<40%) genes are present throughout the virulence plasmid. The low GC-content of these genes suggests that many also encode proteins involved in virulence. Indeed many of these genes encode translocated proteins [16]*.* The virulence plasmid also encodes two autotransporters, one of which, IcsA, is delivered directly into host cells once the *Shigella* are internalized [24]. Because it contains a combination of known translocated and non-translocated proteins the virulence plasmid was initially treated as a “mini genome” to determine the relative sensitivity and specificity of yeast growth inhibition as an indicator of translocated pathogenic proteins.

We expressed three groups of proteins from the Shigella flexneri virulence plasmid in yeast. The first group was composed of 18 proteins translocated by *Shigella* into host cells (Table 1). The second group was composed of 20 proteins that are confined to the bacterium during infection (non-translocated). This group includes proteins involved in plasmid segregation, transcriptional regulation and TTSS chaperones (Table 2). The third group was composed of three candidate translocated proteins based on the low GC-content of their corresponding genes (*orf13*, *orf212* and *ipaJ*). *Shigella* proteins that interact with the bacterial outer membrane as well as the components of the TTSS apparatus were not expressed in yeast because of concern that such membrane associated proteins would not correctly fold when expressed *de novo* in yeast.

**Table 1:**
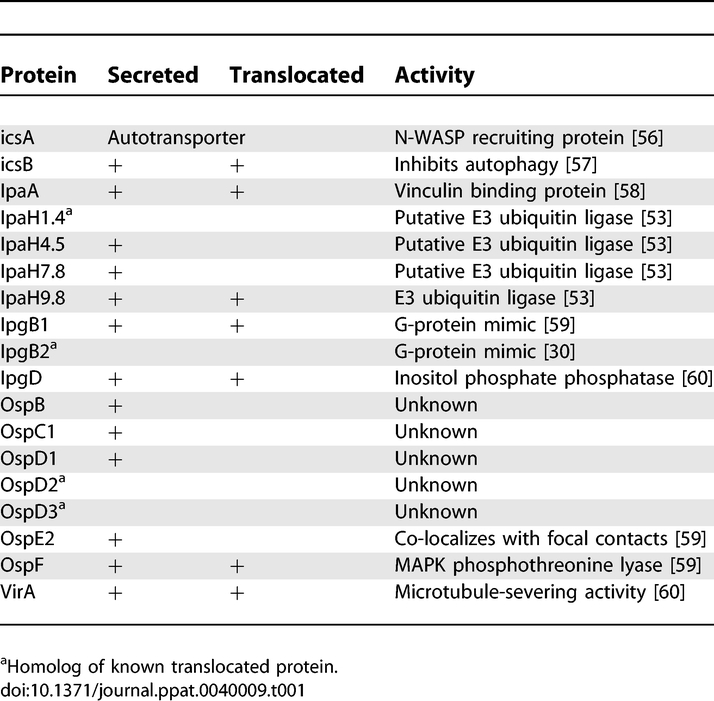
Summary of Known Status of Translocated Proteins

**Table 2:**
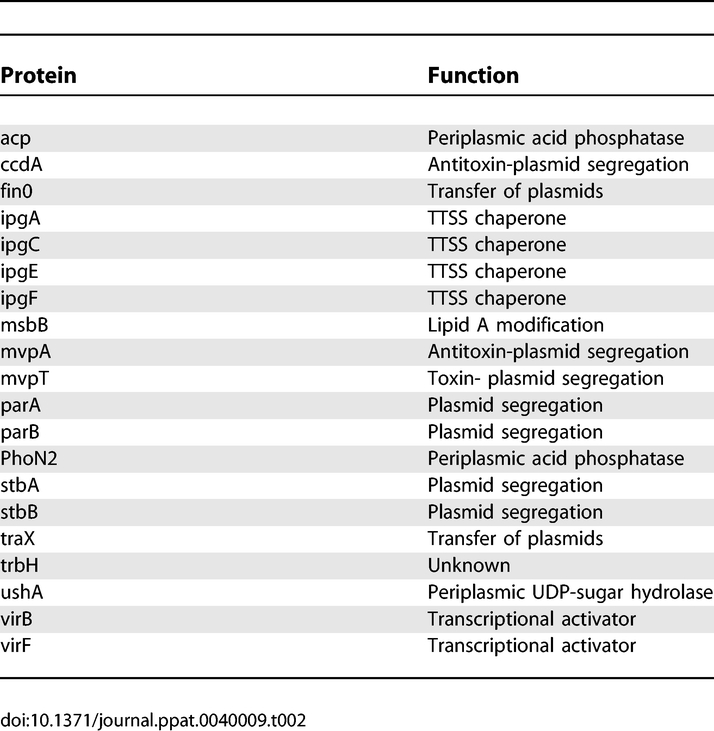
Summary of Functions of Non-translocated Proteins

### Development of a Quantitative Yeast 96 Well Liquid Growth Assay

Several considerations determined the design of our quantitative yeast growth assay. First, heterologous proteins can be introduced and maintained in yeast by integrating their genes into the yeast chromosome, a labor-intensive strategy, or by carrying the genes on a plasmid. Two such plasmids that differ in their mechanism of plasmid maintenance and copy number are commonly used in yeast. The first class of plasmids referred to as 2μ plasmids are maintained at a copy number of 30–50. The second class referred to as centromere (cen) containing plasmids are maintained at a copy number of 1–3. Second, two methods are commonly use to confirm expression of heterologous proteins in yeast. Antibodies can be raised against each protein or proteins can be tagged with a common epitope. Because raising antibodies would be prohibitively laborious in a large screen, we chose the simpler epitope approach. Thus, genes encoding each of the chosen *Shigella* proteins were cloned as GFP fusion proteins on both high and low copy plasmids under the control of an inducible promoter (*GAL10*). GFP was used as the epitope to enable future localization studies.

Traditionally quantitative yeast growth assays involve generating growth curves based on frequent optical density (OD) readings of individual aerated cultures grown in test tubes. Recently it was demonstrated that yeast growth in microscale (350 microliter) liquid cultures parallels that observed in larger cultures [25]. In this assay, a single 96-well plate is incubated at 30°C in a plate reader. The plates are shaken at regular intervals to promote aeration and OD readings are obtained every 20 minutes for 24 hours. This assay produces growth curves very similar to the larger volume methods, but it requires the dedication of a plate reader for 24 hours to a single set of 96 cultures. Alternatively, the commercially available Phenotypic MicroArray technology (Biolog) allows for simultaneous monitoring growth rates from numerous 96-well plates using proprietary technology [4]. However, this technology is relatively costly.

We adapted the microscale method to develop a growth assay to measure the growth of thousands of cultures in parallel. In our assay yeast are incubated at 30°C without agitation in 96-well plates. The OD_600_ of each well is read at 24, 30, 36, 48 and 52 hours, corrected for nonlinearity (Figure S1), and smooth growth curves are spline interpolated through the 5 time points. Although yeast grow more slowly in these non-aerated conditions, the growth curves exhibit the expected lag, exponential, and log phases (Figure S2). Moreover, because the area under the curves is highly correlated with the 48hr time points, a single 48hr measurement actually suffices to assess relative effects. Thus, all subsequent analyses presented herein are based exclusively on the 48hr time points.

### Enrichment of Growth Phenotypes Conferred by Expression of Translocated *Shigella* Proteins

**Figure 1:**
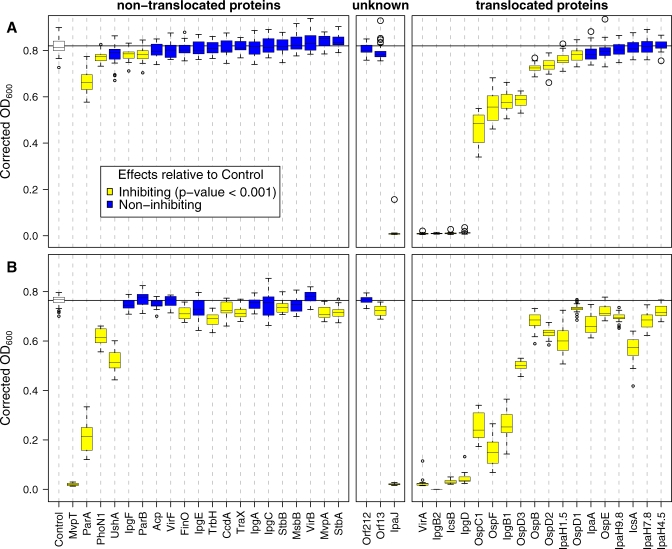
Growth Phenotypes Conferred by Expression of *Shigella* GFP Fusion Proteins in Yeast Each box-and-whisker plot summarizes the OD_600_ measurements of 22 independent yeast cultures expressing the same bacterial protein at t = 48 h. The upper panel shows growth of yeast expressing GFP-*Shigella* fusion proteins encoded on a low-copy number centromere-containing plasmid. The lower panel shows the growth of yeast expressing the GFP-*Shigella* fusion proteins encoded on a high copy number 2μ plasmid. Proteins shown in yellow represent those whose expression resulted in a significant reduction in growth when compared to the control (*p* < 0.001 in a Wilcoxon two-sample test with Bonferroni correction). In the lower panel, "Control" refers to yeast that express low-levels of GFP, while in the upper panel "Control" refers to yeast that express high-levels of GFP. The boxes enclose approximately one quartile either side of the median. The whiskers delimit the ∼95% confidence interval for the mean (using default rendering parameters in the statistical computing software package R [55]). Growth measurements for GFP-MvpT expressed from a yeast low-copy number plasmid are unavailable since we were unable to transform E. coli with this plasmid, which replicates at very high levels in bacteria*.* Since the yeast *GAL1* promotor is weakly active in bacteria, we hypothesize that low-level expression of MvpT, a known bacterial toxin, from a high copy number bacterial plasmid results in a toxic bacterial phenotype.

Figure 1 summarizes the yeast growth phenotypes due to expression of each of the *Shigella* proteins when expressed as a GFP fusion protein from either a low or high copy number plasmid. Under both conditions, expression of the translocated proteins resulted in more frequent and more severe growth inhibition than the non-translocated proteins. The growth phenotypes resulting from expression of the *Shigella* proteins fell into three classes: severe, moderate and minimal to no growth inhibition.

Expression of six *Shigella* proteins completely inhibited yeast growth under all conditions tested. These include four translocated proteins (VirA, IcsB, IpgB2 and IpgD), one non-translocated protein (MvpT), and one protein of unknown secretory status (IpaJ). Expression of four additional translocated proteins (OspC1, OspF, IpgB1 and OspD3) and one non-translocated protein (ParA) conferred intermediate inhibitory growth phenotypes when expressed at low-levels in yeast. Several additional translocated and non-translocated proteins moderately inhibited growth when expressed at high levels.

Overall, low-level expression of the bacterial proteins increased the specificity while high-level expression increased the sensitivity of growth inhibition as an indicator of translocated proteins (Figure 1A vs. 1B). Although, in general, under both conditions tested, expression of the translocated proteins resulted in greater growth inhibition than the non-translocated proteins. Thus, the specificity of the assay for detection of translocated proteins improves when focusing on proteins whose expression inhibits growth more severely.

Expression of GFP-IpgB2 was so toxic to yeast that we were only able to obtain healthy yeast transformants that carry the fusion gene on a low but not a high-copy number plasmid even under conditions that repress the *GAL10* promoter. This is presumably due to low-level basal expression from the *GAL10* promotor in the presence of glucose [26].

### Growth Inhibition Corresponds with Targeting of Conserved Cellular Processes

In the few cases where the activities of the translocated *Shigella* proteins are known, the targets of the proteins that conferred moderate to severe yeast growth inhibition are conserved between yeast and humans. Specifically, VirA [21], IpgD [18], OspF [27–29] and IpgB1 [23] and IpgB2 [30], target microtubules, inositol phosphate signaling, mitogen activated protein kinases and G-protein signaling, respectively. Conversely, the translocated protein IpaA [31], which interacts with vinculin, a protein not present in S. cerevisiae, had no effect on yeast when grown expressed at low-levels and only weakly inhibited yeast growth when expressed at high-level.

Expression of two non-translocated bacterial proteins, MvpT and ParA, also resulted in marked growth inhibition. MvpT is a bacterial toxin that kills bacteria in the absence of its antitoxin, MvpA [32]. Co-expression of MvpA suppressed MvpT toxicity in yeast as it does in bacteria (data not shown). Other bacterial toxin-antitoxin systems have been previously observed to behave similarly in yeast [33,34]. ParA is involved in plasmid partitioning and its close homolog, *Bacillus* Soj, forms nucleoprotein filaments *in vitro* [35]*.* This protein localizes to the yeast nucleus (unpublished data) suggesting that it has a conserved interaction with DNA. Thus, growth inhibition due to MvpT and ParA expression also appear to be due to targeting of conserved cellular processes though in these cases the bacterial proteins likely target basic cellular processes conserved among yeast and prokaryotes.

We were unable to clone *mvpT* onto a low-copy yeast expression plasmid in bacteria without generating mutations in the gene encoding MvpT. However, we were able to clone this gene on to a high-copy number plasmid. Of note the yeast low-copy number plasmid is a high copy number (pUC based, copy number 500–700) bacterial plasmid while the yeast high-copy number plasmid is a low copy number (pBR based, copy number 15–20) bacterial plasmid. Thus, we hypothesize that the low-level expression in bacteria of GFP-MvpT from the *GAL10* promotor or a cryptic bacteria promotor from the high-copy number bacterial plasmid is lethal.

### Growth Inhibition Is a Specific Indicator of Translocated Proteins

Our pilot screen with proteins encoded on the *Shigella* virulence plasmid suggested that yeast growth inhibition is both a sensitive and specific indicator of translocated bacterial proteins. However, this screen only analyzed the effects due to expression of 20 non-translocated proteins. Since these non-translocated proteins are encoded on a virulence plasmid, there was an inherent bias in this screen towards bacterial proteins involved in plasmid maintenance/segregation and regulation of type III secretion. This bias might overestimate the specificity of yeast growth inhibition for detection of translocated proteins since it is conceivable that bacterial housekeeping proteins involved in cellular processes highly conserved among prokaryotes and eukaryotes might act as dominant negative alleles to inhibit yeast growth. For example, expression of *Legionella* sterol desaturase, a homolog of S. cerevisiae ERG25 an essential protein involved in ergosterol biosynthesis [36], results in yeast growth inhibition [8]. Thus, we set out to systematically examined the effects on yeast growth due to a comprehensive collection of ORFs encoded within a bacterial genome by taking advantage of the availability of a library of annotated Francisella tularensis subspecies *tularensis* (strain SCHU S4) (F. tularensis) ORFs cloned into a recombination-based cloning vector [37,38].

F. tularensis is the causative agent of tularemia. Like *S. flexneri, F. tularensis* is a gram-negative intracellular pathogen. Based on comparative genomic analyses and extensive pathway prediction modeling, a putative function has been assigned to approximately two-thirds of the ∼1,600 annotated F. tularensis ORFs [39]. These include ORFs involved in metabolism, transcription, and translation. Since *Francisella* encodes homologs of common bacterial housekeeping genes, a survey of the behavior of *Francisella* proteins in yeast should provide insight into how other bacterial genomes would behave when expressed in yeast.

Based on the results of our pilot screen, we decided to transfer each *Francisella* ORF to a low-copy number yeast expression vector such that each ORF was expressed as a GFP fusion protein under the control of the *GAL1* promotor. We constructed a yeast expression plasmid compatible with the Gateway recombination-based system to facilitate transfer of the sequence-verified *Francisella* ORFs. Each of the translocated *Shigella* proteins conferred similar effects on yeast growth when expressed from either the original “traditional” low-copy number yeast expression plasmid or the newly constructed recombination-based cloning vector (Figure S3). We successfully transferred approximately two-thirds of the annotated F. tularensis ORFs into a yeast expression plasmid. Each ORF was transformed into yeast and up to six biologic replicates were analyzed for growth inhibition under the same conditions used to analyze yeast expressing the *Shigella* proteins (Table S1).

**Figure 2:**
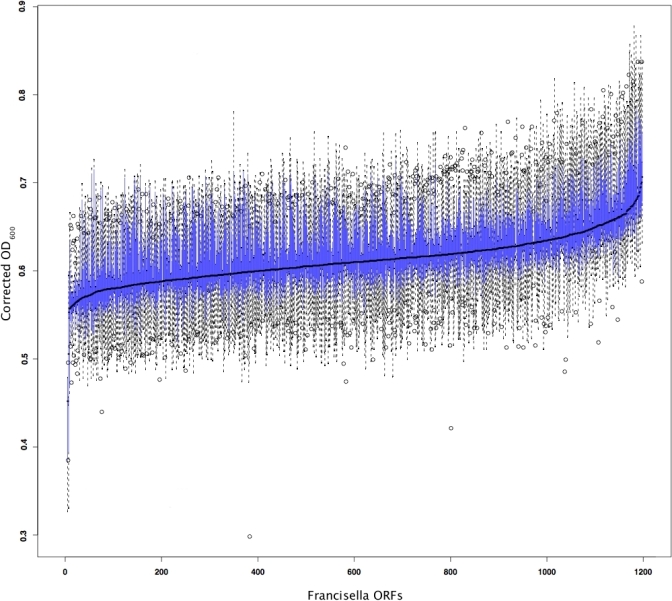
Growth Phenotypes Conferred by Expression of *Francisella* GFP Fusion Proteins in Yeast Each box-and-whisker plot summarizes the OD_600_ measurements of six independent yeast cultures expressing the same bacterial protein at t = 48h. Yeast strains expressing each of the proteins are ordered by their mean OD_600_ reading. The boxes enclose approximately one quartile either side of the median. The whiskers delimit the ∼95% confidence interval for the mean (using default rendering parameters in the statistical computing software package R [55]). A blow-up of the first 100 yeast strains displayed in this graph is displayed in Figure S4.

Expression of none of the *Francisella* proteins screened severely inhibited yeast growth and expression of only three *Francisella* proteins resulted in minimal but reproducible growth inhibition (Figures 2 and S4). These proteins include FTT1130c (CphA, cyanophycin synthetase), FTT0396 (ParC, topoisomerase IV subunit A) and FTT0384c (a phosphatidylserine decarboxylase (*psd*)). *parC* and *psd* are likely housekeeping genes and were recently identified as essential F. novicida genes in a transposon library screen [40]. Cyanophycin synthetase is involved in synthesis of cyanophycin, a non-ribosome amino acid polymer common to cyanobacteria. The significance of the presence of this compound in *Francisella* is currently unknown.

We next examined the levels of expression of the bacterial GFP fusion proteins in yeast to determine whether the lack of growth inhibition was due to poor expression of the *Francisella* proteins. The majority of the 48 *Francisella* and 39 *Shigella* proteins examined were expressed at comparable levels (Figure S5). However, expression of approximately 33% of the *Francisella* proteins and 15% of the non-toxic *Shigella* proteins was undetectable (expression of three of the five toxic translocated proteins was undetectable presumably due to their associated severe growth inhibition). The significance of the decreased level of expression of the *Francisella* proteins is unclear, but may reflect their relative decreased G-C content (32.6%) as compared to the *Shigella* (39.8%) and endogenous yeast proteins (38.3%). Alternatively, if we assume that all of the toxic proteins are expressed in yeast, then all but two of the translocated proteins (89%) were expressed in yeast, which suggests that these proteins have been selected for stability in eukaryotic cells. Thus, the relative decreased stability of the *Francisella* proteins might be due to optimization of their stability in prokaryotic as compared to eukaryotic cells.

In regards to the sensitivity of growth inhibition for detection of translocated *Francisella* proteins, the significance of the scarcity of yeast growth inhibition is unclear. There is currently no data to demonstrate that F. tularensis secrete proteins directly into host cells [41]. These bacteria do not appear to encode type III, type IV or type V secretion systems. However, some species of *Francisella* secrete proteins via type IV pili [42] (although there is no evidence that this secretion system is functional in F. tularensis) and F. tularensis does appear to encode a type VI secretion system [43–45]. Since we have only screened approximately 2/3 of the annotated *Francisella* ORFs in yeast it is possible that a more complete screen would identify potential translocated proteins. Similarly, it is possible that translocated *Francisella* proteins are expressed too poorly in yeast to interfere with yeast growth and that increased levels of expression from a high copy number plasmid might result the sensitivity of detection of translocated F. tularensis proteins.

Nevertheless, the rarity of growth inhibitory phenotypes observed with expression of both F. tularensis and non-translocated S. flexneri proteins in yeast demonstrates that yeast growth inhibition is a rare event and not due to random overexpression of bacterial proteins. Furthermore, since ∼50% of *Francisella* ORFs have E. coli homologs [40] it seems likely that proteins from other gram-negative will behave similarly when expressed in yeast. Thus, growth inhibition appears to be a highly specific indicator of translocated proteins.

### The Addition of Stressors Increases Detection of Translocated Proteins

Presumably, the targets of some translocated proteins are not conserved among yeast and mammals and thus expression of these proteins will not affect yeast growth. Alternatively, a translocated protein may target a pathway that is conserved but is not normally rate-limiting for yeast growth. To address the latter possibility, yeast expressing all 42 *Shigella* GFP fusion proteins at low-levels were grown in the presence of four well-characterized yeast stressors: salt, nocodazole, sorbitol and caffeine [46]. Each was administered at levels that minimally impact wild-type growth. High salt and sorbitol both increase osmotic stress while high salt also results in a disturbance in ion homeostasis, nocodazole, destabilizes microtubules and caffeine has pleiotropic affects on yeast including inhibition of MAP kinases and calcium channels.

**Figure 3:**
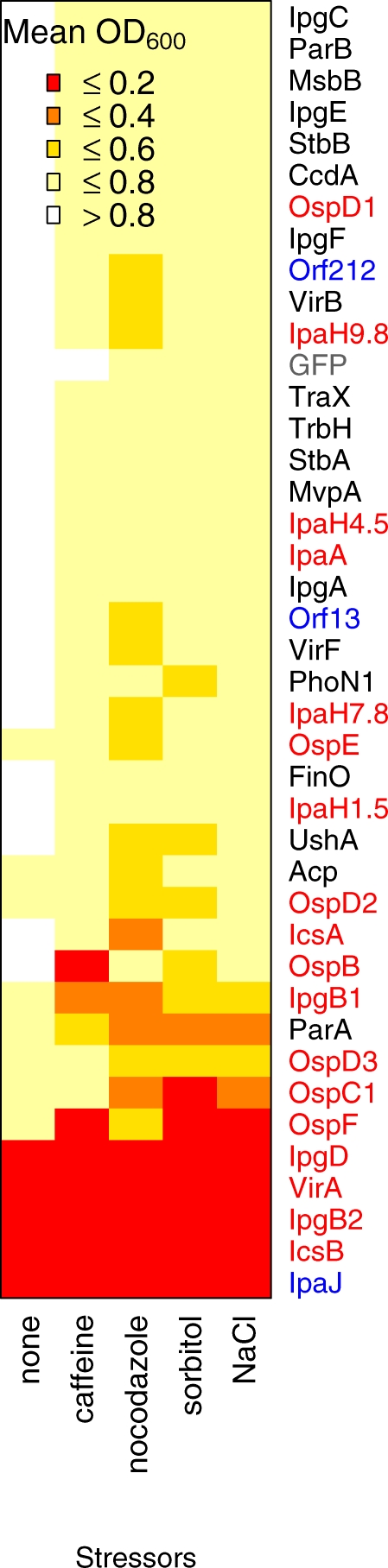
The Addition of Stressors Elicits Additional Yeast Growth Inhibition Phenotypes by Translocated Proteins The heatmap summarizes the relative growth at t = 48 h of yeast that express the *Shigella* GFP fusion proteins or GFP grown under each of five conditions: no stressor, 6 mM caffeine, 3 μg/ml nocodazole, 0.5 M sorbitol, and 0.5M NaCl. Each cell represents the mean OD_600_ measurements at 48 h of six independent yeast colonies expressing the same bacterial protein from a low-copy yeast expression plasmid grown under the same condition. Rows and columns were clustered by the *complete linkage* algorithm using the standard Euclidean distance metric operating directly on data vectors (i.e., without normalization). The proteins labeled in red are translocated, those labeled in black are non-translocated, and the unknowns in blue. GFP control is labeled in gray.

The addition of the stressors to the media elicited marked changes in the phenotypes of two translocated proteins (Figure 3). OspB weakly inhibited growth in standard conditions, but in the presence of caffeine low-level OspB expression severely inhibited yeast growth. The phenotype of IcsA was similarly amplified by the addition of nocodazole. Although these were the only strictly conditional phenotypes, yeast expressing proteins that conferred intermediate growth phenotypes when expressed at low-levels (IpgB1, OspC1, OspD3, OspF and ParA) displayed different sensitivities to the four stressors, implying that the phenotypes conferred by each of these proteins is due to a specific activity of the protein. In summary, the stress conditions increased the sensitivity of the assay with no loss of specificity.

We assayed for growth inhibition in the presence of four well-characterized yeast stressors; however, there are numerous other agents that could also be applied. The potential value of such assays goes beyond the identification of proteins whose targets are not limiting for growth. The patterns of sensitivities of yeast expressing the bacterial proteins to individual stressors might provide insights into cellular processes targeted by the proteins. For example, the sensitivities of the complete set of ∼4,700 viable yeast deletion strains to dozens of stressors have been measured [46,47]. Thus, it should be possible to compare sensitivity patterns of the yeast deletion strains and yeast expressing bacterial proteins. The yeast genome is one of the most exhaustively characterized of any organism, so similarities in the patterns of sensitivities to various stressors between yeast deletion strains and wild type yeast expressing a given bacterial protein can potentially provide insight into the activity of the bacterial proteins. Such strategies have been successfully applied to identify the cellular targets of therapeutic agents [47,48].

### Fusion to GFP Generally Exacerbates Rather Than Masks Growth Inhibition

**Figure 4:**
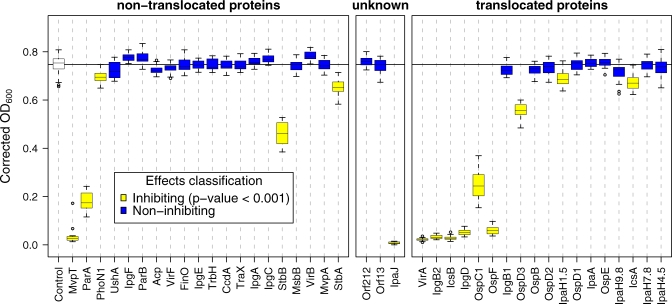
Growth Phenotypes Conferred by Expression of *Shigella* Proteins in Yeast Each box-and-whisker plot summarizes the OD_600_ measurements of 22 independent yeast cultures expressing a single *Shigella* protein at t = 48 h. Proteins shown in yellow represent those whose expression resulted in a significant reduction in growth when compared to the control (*p* < 0.001 in a Wilcoxon two-sample test with Bonferroni correction). “Control” refers to yeast that carry empty vector. The boxes enclose approximately one quartile either side of the median. The whiskers delimit the ∼95% confidence interval for the mean (using default rendering parameters in the statistical computing software package R [55]).

Another explanation for the lack of detection of yeast growth inhibition due to expression of bacterial proteins is that fusion to GFP could potentially interfere with the activity and/or folding of the proteins and thus mask phenotypes. To address this possibility we conducted growth assays on yeast that express the *Shigella* proteins in the absence of GFP. The absence of GFP decreased the overall toxicity of almost all the *Shigella* proteins when expressed from a high-copy number plasmid (Figure 4 vs. Figure 1). High-level expression of all the *Shigella* GFP fusion proteins inhibited growth more often than their non-GFP counterparts (75% vs. 38%).

In general, proteins that conferred the weakest growth phenotypes when fused to GFP and expressed at high-levels, no longer resulted in significant growth inhibition when expressed on their own or when expressed as GFP fusion proteins at relatively low-levels in yeast. In the extreme case, IpgB1, a strong growth inhibitor when fused to GFP, conferred no detectable inhibition when expressed without GFP. Nevertheless, fusion to GFP appears to interfere with the activity of StbB and OspF, though only in the case of StbB was the growth inhibition phenotype entirely masked. Overall, these observations suggest that, in general, fusion to GFP increased the stability of the fusion proteins [49] and/or the transcriptional efficiency of the genes encoding the *Shigella* fusion proteins without disrupting their function.

### IpaJ Is a Substrate of the *Shigella* Type III Secretion System

**Figure 5:**
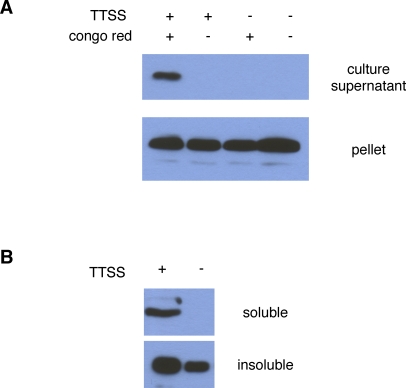
IpaJ Secretion and Translocation Are Dependent on an Intact Type III Secretion System (A) Wild-type and BS547 (*mxiM1*) [50] *Shigella,* a TTSS-defective strain, that carry a low-copy number plasmid that expresses IpaJ-FLAG from its endogenous promotor were grown in the presence of Congo Red, an inducer of the *Shigella* TTSS. Proteins from culture supernatants and whole-cells were separated by SDS-PAGE and subjected to western blot analyses with anti-FLAG antibody. (B) HeLa cells were infected with either wild-type or BS547 (*mxiM1*) *Shigella* that express IpaJ-FLAG at an MOI of 100:1. One hour after infection, the cells were lysed and fractionated into soluble and insoluble fractions. Proteins from each fraction were separated by SDS-PAGE and subjected to western blot analyses with anti-FLAG antibody. Translocated proteins are found in the soluble fraction, while those from unlysed bacteria are found in the insoluble fraction.

Of the three proteins of unknown function we expressed in yeast, only one, IpaJ, resulted in yeast growth inhibition. To determine if we had identified a new substrate of the *Shigella* TTSS, a plasmid that encodes IpaJ fused to 3x FLAG tag was introduced into wild type *Shigella* strain as well as a *Shigella* strain defective in TTSS due to a mutation in *mxiM1* [50], a component of the secretion apparatus. As demonstrated in Figure 5A, IpaJ is only detected by western blot analyses in the supernatants of wild-type *Shigella* after the addition of Congo red, a TTSS inducer, to the media [51]. Similarly, as shown in Figure 5B, detectable levels of IpaJ within host cells during the course of an infection is also dependent on an intact TTSS. Secretion of IpaJ appeared to be ∼100 times lower than that of either IpgB1 or OspF (data not shown). This difference in secretion levels may explain the lack of detection of IpaJ in prior analyses of native proteins in supernatant preparations of *Shigella* mutants that constitutively secrete effector proteins [16]. Thus, our growth inhibition screen led to the identification of a new effector protein that had been previously missed by other experimental approaches.

### Summary

We have developed a semi-automated quantitative yeast assay to measure the growth of thousands of strains in parallel. Using this system, we demonstrate that yeast growth inhibition is a relatively specific and sensitive indicator of translocated bacterial proteins that target host cells. The efficiency of our growth assay combined with the increasing availability of bacterial genomes in recombination-based cloning vectors [52] which are easily transferred to yeast expression vectors, makes this assay a compelling functional genomic screen for the identification of translocated proteins. In particular, because it only requires DNA, the yeast growth assay is invaluable for studying organisms difficult or dangerous to grow. Furthermore, once a library of yeast expressing bacterial proteins is generated, additional high throughput assays can be designed to screen for bacterial proteins that target specific conserved eukaryotic cellular pathways. For example, Shohdy and colleagues used a reporter plasmid to screen for Legionella pneumophila proteins that interfere with yeast membrane trafficking [10]. Similarly, Rhode and colleagues identified a *Shigella* effector, IpaH9.8 that specifically inactivates the yeast mating pathway, a highly conserved MAPK signaling pathway, by targeting a mitogen activated kinase kinase for degradation via ubiquination [53]. Lastly, prior work has established that detailed understanding of a protein's activity in yeast can provide insights into its activity in more complex organisms [7–13]. Thus, bacterial proteins that inhibit yeast growth are obvious candidates for future studies in yeast to determine the molecular mechanisms underlying growth inhibition.

## Materials and Methods

### Plasmids and strains.

The *Shigella* genes cloned into the yeast expression vectors are summarized in Table S2. Each designated open reading frame was PCR amplified from a 2457T *S. flexneri* serotype 2a DNA preparation. Genes were subcloned into two high copy number (2μ) plasmids (copy number 30–50) that carry the *LEU2* gene and the *GAL10* promoter, pFUS-GFP and pFUS-HIII [7]. The genes were either cloned in-frame with the carboxy-terminus of GFP (pFUS-GFP) or in-frame with a short sequence between the pFUS-HIII start codon and polylinker. The primers used for amplification via the polymerase chain reaction (PCR) contained engineered restriction sites at their 5′ (*ApaI* or *XhoI*) and 3′ (*NheI*) ends. When the *ApaI* site was introduced, the bacterial gene ATG was converted to CTG, thus converting methionine to leucine. The *icsB* gene, which contains an *NheI* site, was cloned directly into the pFUS vectors by homologous recombination in yeast. Each of the PCR-amplified genes was analyzed by DNA sequencing. The sequences were compared to the sequence of the Shigella flexneri serotype 2a strain 2457T virulence plasmid (generously provided by Dr. Val Burland (University of Wisconsin)). With the exception of two synonymous mutations all of the sequence changes had been previously observed. Sequence variations specific to the 2457T virulence plasmid as compared to the published *Shigella* virulence plasmids are summarized in Table S2. Low-copy-number (centromere-containing) plasmid (copy number 1–3) versions of the clones were constructed by homologous recombination gap repair into pRS313 that contains the *GAL10* promotor/GFP and terminator regions of the pFUS plasmid.

The sequence-verified *Francisella* genes present in the Gateway pDNR entry vector were transferred to pBY011-GFP-clonNAT by a Gateway LR reaction. pBY011-GFP-clonNAT is a derivative of pBY011-D123. The gene encoding GFP was introduced downstream of the *GAL1–10* promotor and upstream of the attR1 site at the *XbaI* site. The clonNAT gene was introducing at the site in pBY011-D123. All yeast expression plasmids were transformed into wild-type yeast strain S288C (BY4741 MAT**a**) using the PEG/Lithium acetate method.

### Yeast growth assays.

Growth phenotypes of yeast that conditionally express the *Shigella* proteins were assayed in 96-well plates. Individual yeast transformants were inoculated into each well of a 96-well plate containing non-inducing selective synthetic media supplemented with 4% glucose. Saturated cultures were transferred to non-inducing selective synthetic solid media, 4% glucose using a 3.18 mm diameter 96-pinner tool (V&P Scientific, Inc., San Diego, CA) on a Biorobot 3000 (Qiagen, Valencia, CA). After one to two days, the yeast spots on the solid plates were transferred to fresh liquid 96-well plates using a 1.58 mm diameter 96-pinner tool and incubated 16–18 hours to grow to OD_600_ between 0.3–0.4. The liquid cultures were next transferred with 1.58mm diameter pinner to inducing selective synthetic liquid media containing 4% galactose in 96-well plates (“induction plates”). All incubations were done at 30°C and in the absence of agitation. In the case of the stressors, selective synthetic liquid media containing 4% galactose was supplemented with 500 mM sodium chloride, 6 mM caffeine, 0.5 M Sorbitol, and 3 μg/ml nocodazole. Growth in the 96-well plates was monitored by OD_600_ readings with a Wallac Victor II multiplate reader (Perkin Elmer, Shelton, CT) at 24, 32, 40, 48 and 52 hours. The plates are shaken prior to being read in plate reader to allow for even distribution of colonies in the media.

### Screening for yeast growth inhibition due expression of F. tularensis proteins.

Thirteen hundred and seven individual annotated F. tularensis Schu 4 sequence-verified open reading frames were transferred from a Gateway entry vector to pBY011-GFP-clonNAT, a yeast expression plasmid, using Gateway technology (Invitrogen). Thirty-seven of these reactions failed to yield bacterial transformants and were dropped from the screen. Plasmids were subsequently isolated from the remaining 1270 transformants. On two separate occasions, DNA from these transformants was transferred into wild type yeast (S288C) using a yeast 96-well transformation protocol using a 3.18 mm diameter 96-pinner tool. In each case, the transformants were selected on three independent 96-well plates, thus a total of potentially six independent yeast transformations were examined for each clone. A 96-pinner device with 1.58 mm pins was then used to transfer a subset of the transformants to a second solid media SC-URA non-inducing tray to allow for “normalization” of the amount of yeast recovered from each transformation. Yeast containing each of the *Francisella* ORFs were subjected to the 96 well liquid growth assay described above.

As summarized in Table S1 the majority of the plasmids (1116) were successfully transformed in each of the six biologic replicates. In the 165 cases where six transformants were not obtained, the plasmids used to transform the yeast were examined by gel electrophoresis. Of these 165 plasmids, 61 were observed to have undergone an incorrect recombination event during the Gateway reaction and thus were classified as additional Gateway failures. We then restriction digested 50 of the plasmids used in the successful yeast transformations. Five of these plasmids demonstrated incorrect restriction patterns suggesting that ∼10% of the remaining Gateway reactions were incorrect. Thus, we estimated that we have successfully screened for growth phenotypes due to expression of ∼1,000 F. tularensis ORFs.

Several observations suggest that the lack of detection of *Francisella* proteins that inhibit yeast growth is not due to false negatives. First, we did initially identify four *Francisella* ORFs that appeared to significantly inhibit yeast growth. Thus, our screen was indeed capable of identifying yeast strains impaired in growth. However, upon further analysis, it was apparent that the plasmid introduced into these strains, also resulted from an incorrect recombination event such that the plasmid no longer encoded a *Francisella* ORF. Indeed this plasmid was smaller than expect and yeast carrying this plasmid were impaired for growth under both inducing (galactose) and non-inducing (glucose) conditions. Second, based on the behavior of the known blanks and gateway recombination failures there was a low-rate of cross-contamination. Only 12.9% of the expected blank spots showed any growth. Thus, it is extremely unlikely that >1 of the 6 transformants of each *Francisella* ORFs screened was contaminated. Furthermore, examination of the original transformation plates demonstrates that when there is a contaminant, the most likely source is at the time of transformation and the rate of contaminant is minimal.

### Yeast western blot assays.

Yeast that conditionally expressed each of the *Shigella* translocated and non-translocated proteins studied as well as 48 *Francisella* proteins fused to GFP were grown overnight in selective media supplemented with 2% raffinose. In the AM, cultures were back-diluted to OD_600_ = 1.0 in selective media supplemented with 2% raffinose. After 2 hours, protein production was induced by the addition of galactose to a final concentration of 4%. After an additional 4 hours, the yeast were pelleted and quickly frozen in a dry-ice/ethanol bath. The yeast were subsequently lysed using a bead-beat protocol and equal volumes of samples were analyzed by SDS-PAGE and subjected to western blot analyses with anti-GFP polyclonal antibody (Sigma). PSTAIRE antibody (Santa Cruz Biotechnology) was used to monitor levels of Cdc2 as a loading control.

### Secretion and translocation assays.

A “sewing” PCR reaction was used to fuse IpaJ plus its upstream 500 nucleotides to a triple FLAG tag at its carboxy terminus. This fusion gene was cloned into pAM238 (pSC101 ori) and the resulting plasmid was introduced into wild-type and BS547 (*MxiM::aphA-3*) [50] 2457T Shigella flexneri serotype 2a. To determine if IpaJ-FLAG was secreted by the *Shigella* strains, overnight cultures grown in TCS (trypticase soy) broth were diluted 1:100 into 6 mls TCS broth. The diluted cultures were grown for 2 hours until they reached an OD_600_ of ∼0.5. The bacteria were resuspended in 2 mls phosphate-buffered saline (PBS) containing 10 uM Congo red (CR) (Sigma) and incubated for an additional 30 minutes. All incubations were conducted at 37°C. After this incubation the bacteria were centrifuged and the pellets were saved for the whole-cell lysates. The supernatant was then subjected to a second centrifugation step to ensure that few *Shigella* were present in the supernatant fraction. Proteins present in the second supernatant were precipitated by the addition of TCA (trichloroacetate). Proteins were analyzed by Western blotting with an anti-FLAG antibody (Sigma).

For translocation assays, HeLa cells were infected (MOI of 1:100) for 1 hour with wild-type and BS547 (*MxiM::aphA-3*) *Shigella* that express IpaJ-FLAG. To synchronize infections and increase the efficiency of invasion, a plasmid that expresses, the plasmid pIL22 that constitutively expresses the afimbrial adhesin from uropathogenic E. coli was introduced into the *Shigella* strains [54]. Cells were washed with ice cold PBS and then lysed with 300 ul RIPA buffer plus protease inhibitors. The cells were spun down and the pellet was designated the insoluble fraction. The supernatant was spun down a second time and the result supernatant was designated the soluble fraction. Equal volumes of soluble and insoluble fractions were subjected to SDS-PAGE. Gels were blotted to nitrocellulose and probed with the anti-FLAG antibody (Sigma).

## Supporting Information

Figure S1 Correction of OD_600_ Readings at High Densities A series of dilution measurements was conducted to derive a correction equation to correct for the loss of linearity of OD_600_ readings at higher yeast culture densities (A). The mean value of eight separate dilutions at each concentration level was taken to represent OD_600_ at that dilution, and a line was fit by simple regression to the first nine data points judged by eye to represent the most linear range. The dotted line maps concentration, *c*, to “correct” optical density, *d*_cor_, simply extrapolating the linearity of low concentrations into the high range.
f:c → d_cor_ where f(x) = a_1_x + a_0_
A cubic polynomial was fit by least squares regression to the entire 25 data points. For convenience in forming the function composition that yields our correction equation, the fit polynomial is actually the inverse of that depicted. That is it maps the observed density (the *y*-axis) to concentration (the *x*-axis).
g:d_obs_ → c where g(x) = b_3_x^3^ + b_2_x^2^ +b_1_x+b_0_
The correction equation is then simply the composition of these two functions:
h = f(g):d_obs_ → d_cor_ where h(x) = 0.453x^3^ − 0.230x^2^ + 1.009x−0.002
Finally, the OD_600_ of empty media is subtracted to zero the result yielding:
h(x) = 0.453x^3^ – 0.230x^2^ + 1.009x−0.035
(B) The correction curve is depicted along with the y = x curve. The correction curve crosses y = x at 0.657.
(31 KB PDF)

Figure S2 Growth Curves of Yeast Expressing High Levels of *Shigella* GFP Fusion Proteins in 96-Well Plates Growth assays were conducted on 22 independent yeast transformants that each conditionally express one of the GFP-*Shigella* fusion proteins. The OD_600_ of each well was read at t = 24, 32, 40, 48, and 52 h after the induction of expression of the fusion proteins. The growth curves were spline-interpolated from the mean OD_600_ at each of the five time points.
(36 KB PDF)

Figure S3 Yeast Growth Phenotypes Conferred by Low-Level Expression of *Shigella* GFP Fusion Proteins from a Gateway-Derived Vector Each box-and-whisker plot summarizes the OD_600_ measurements of six independent yeast cultures expressing the same bacterial protein at t = 48 h. The boxes enclose approximately one quartile either side of the median. The whiskers delimit the ∼95% confidence interval for the mean (using default rendering parameters in the statistical computing software package R [55]).
(34 KB PDF)

Figure S4 Growth Phenotypes Conferred by Expression of *Francisella* GFP Fusion Proteins in Yeast This blow up displays the first 100 yeast strains expressing the *Francisella* proteins when ordered by their mean OD_600_ reading. Each box-and-whisker plot summarizes the OD_600_ measurements of six independent yeast cultures expressing the same bacterial protein at t = 48 h. The boxes enclose approximately one quartile either side of the median. The whiskers delimit the ∼95% confidence interval for the mean (using default rendering parameters in the statistical computing software package R [55]).
(298 KB PDF)

Figure S5 Expression of *Shigella* and *Francisella* Proteins in Yeast Overnight cultures of yeast that conditionally express GFP fused to each of the 19 translocated and 20 non-translocated *Shigella* proteins studied as well as 48 of the *Francisella* proteins were grown under non-inducing conditions (2% raffinose). In the AM, expression of the recombinant proteins was induced by the addition of 4% galactose to exponentially growing cultures for 4 h. Total protein was extracted and resolved by SDS-PAGE. The proteins were analyzed by western blot analyses using polyclonal anti-GFP antibody (BD Living Colors full-length A. v. polyclonal Ab) (Clontech). A white dot is shown next to the approximate size of the expected GFP fusion protein. Levels of Cdc2 p34 (PSTAIRE) were monitored as a loading control (Santa Cruz Biotechnology, Inc.).
(9.4 MB PDF)

Table S1 Summary of Growth Phenotypes Resulting from Expression of *Francisella* Proteins in Yeast (276 KB XLS)

Table S2 Summary of Nucleotide and Amino Acid Changes in Cloned Shigella flexneri Genes/Proteins (35 KB DOC)

### Accession Number

The GenBank accession number for the *Shigella* virulence plasmid that encodes both the translocated and non-translocated proteins is gi|31983523:106938.
