# Supplementary material for: A Functional Genomic Yeast Screen to Identify Pathogenic Bacterial Proteins
Source: PLoS Pathog. 2008 Jan 18;4(1):e9. doi: 10.1371/journal.ppat.0040009 (PMC2211553; doi:10.1371/journal.ppat.0040009)
Supplement: Supplementary file 1 [file ppat.0040009.sg001.pdf]

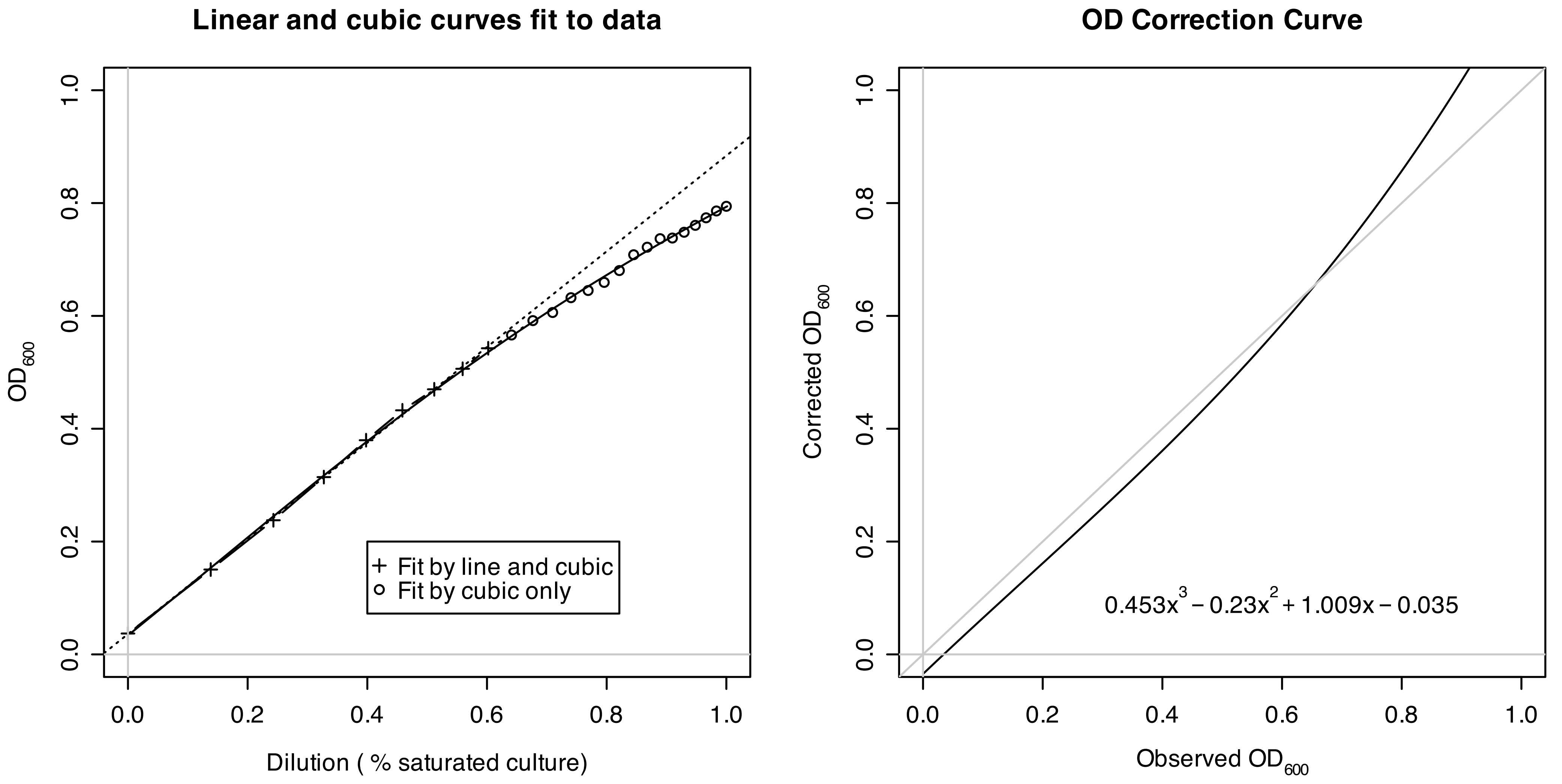

Linear and cubic curves fit to data
OD Correction Curve
OD_{600}
Dilution (% saturated culture)
Fit by line and cubic
Fit by cubic only
Corrected OD_{600}
Observed OD_{600}
$0.453x^3 - 0.23x^2 + 1.009x - 0.035$
0.0
0.2
0.4
0.6
0.8
1.0
