# Supplementary material for: A Functional Genomic Yeast Screen to Identify Pathogenic Bacterial Proteins
Source: PLoS Pathog. 2008 Jan 18;4(1):e9. doi: 10.1371/journal.ppat.0040009 (PMC2211553; doi:10.1371/journal.ppat.0040009)
Supplement: Supplementary file 2 [file ppat.0040009.sg002.pdf]

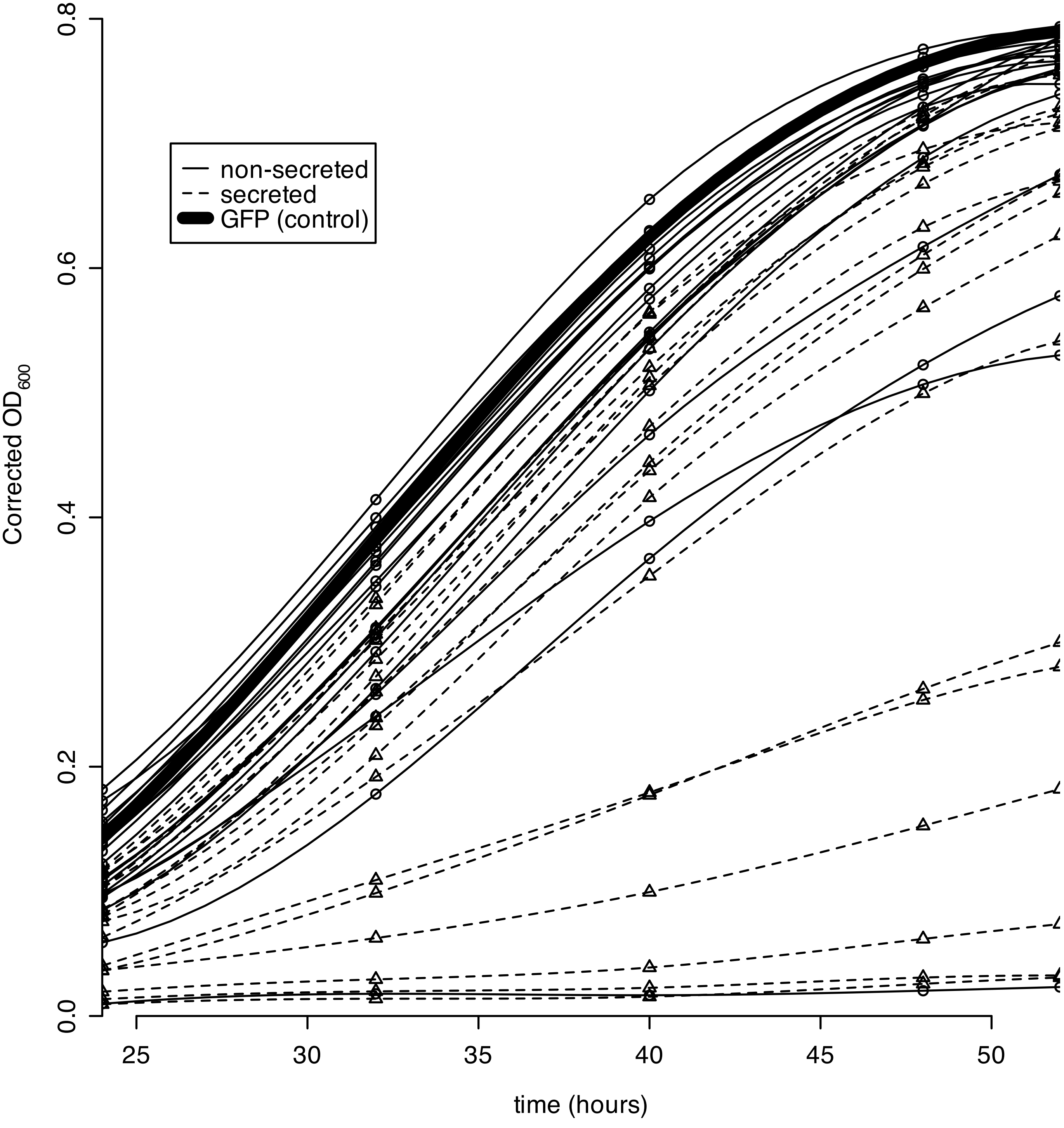

non-secreted
secreted
GFP (control)
Corrected OD_{600}
time (hours)
0.0
0.2
0.4
0.6
0.8
25
30
35
40
45
50
