# Supplementary material for: A Functional Genomic Yeast Screen to Identify Pathogenic Bacterial Proteins
Source: PLoS Pathog. 2008 Jan 18;4(1):e9. doi: 10.1371/journal.ppat.0040009 (PMC2211553; doi:10.1371/journal.ppat.0040009)
Supplement: Supplementary file 3 [file ppat.0040009.sg003.pdf]

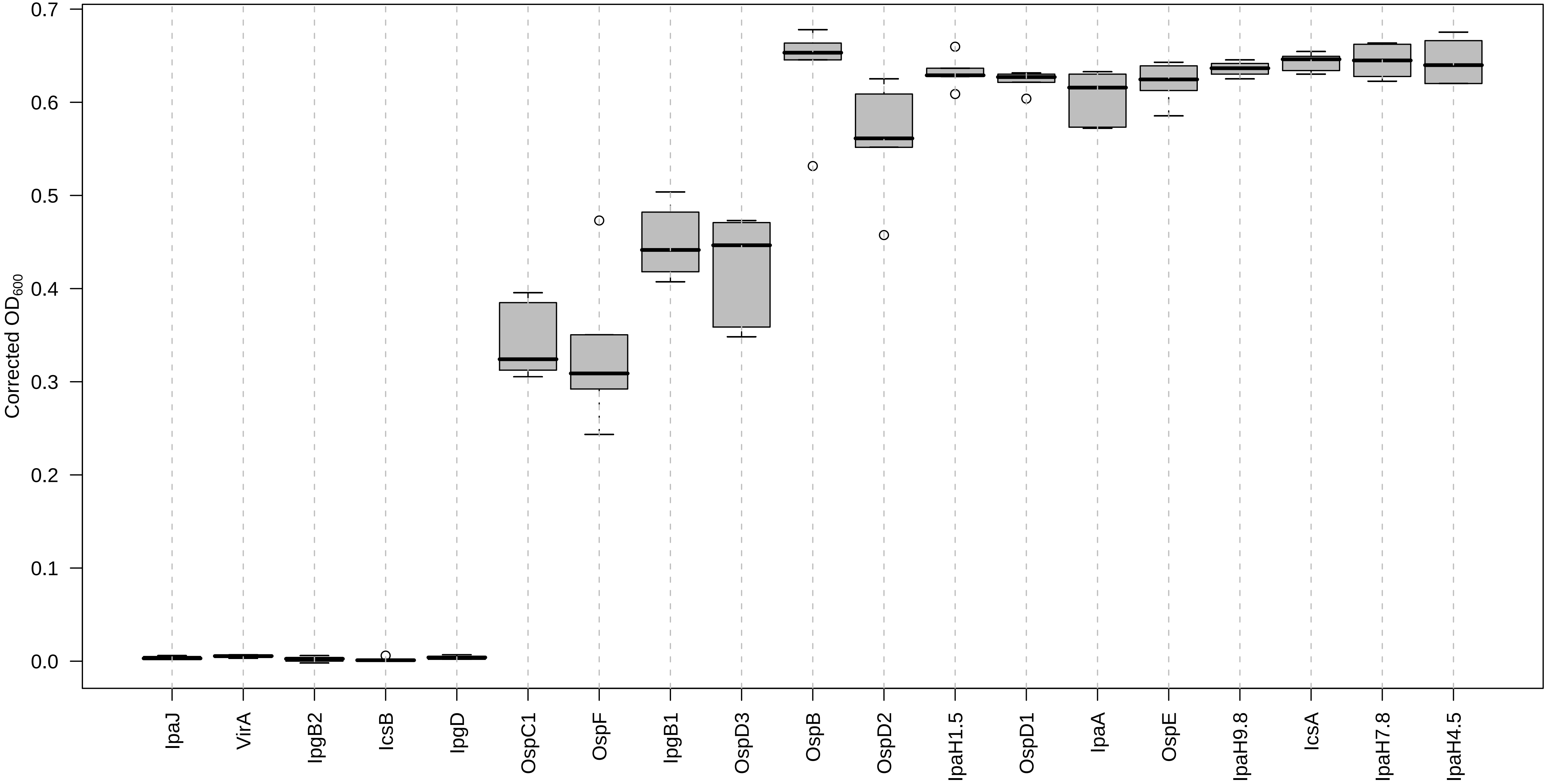

Corrected OD600
0.0
0.1
0.2
0.3
0.4
0.5
0.6
0.7
IpaJ
VirA
IpgB2
IcsB
IpgD
OspC1
OspF
IpgB1
OspD3
OspB
OspD2
IpaH1.5
OspD1
IpaA
OspE
IpaH9.8
IcsA
IpaH7.8
IpaH4.5
