# Supplementary material for: A Functional Genomic Yeast Screen to Identify Pathogenic Bacterial Proteins
Source: PLoS Pathog. 2008 Jan 18;4(1):e9. doi: 10.1371/journal.ppat.0040009 (PMC2211553; doi:10.1371/journal.ppat.0040009)
Supplement: Supplementary file 4 [file ppat.0040009.sg004.pdf]

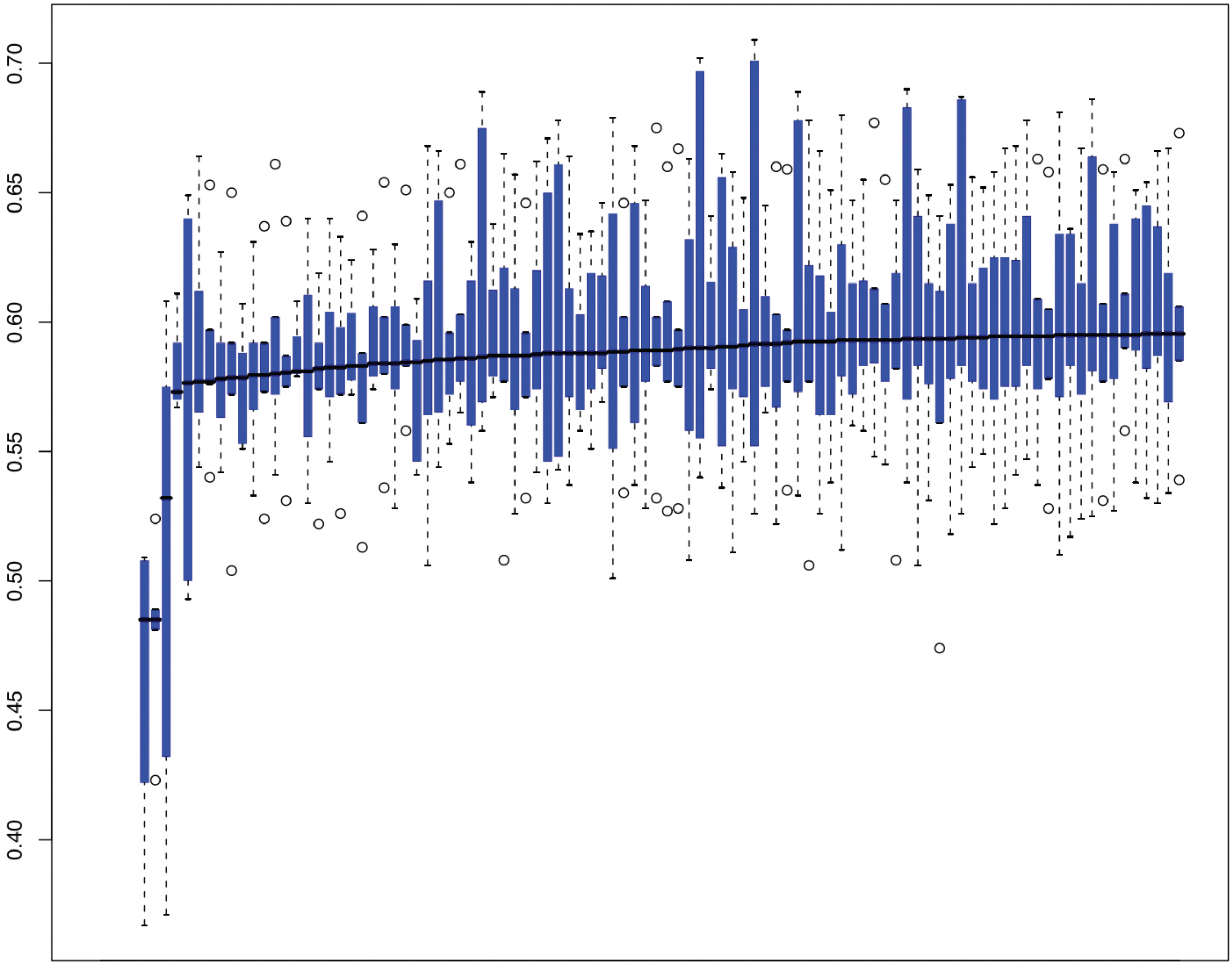

0.40
0.45
0.50
0.55
0.60
0.65
0.70
