# Supplementary material for: A Functional Genomic Yeast Screen to Identify Pathogenic Bacterial Proteins
Source: PLoS Pathog. 2008 Jan 18;4(1):e9. doi: 10.1371/journal.ppat.0040009 (PMC2211553; doi:10.1371/journal.ppat.0040009)

non-translocated *Shigella* proteins

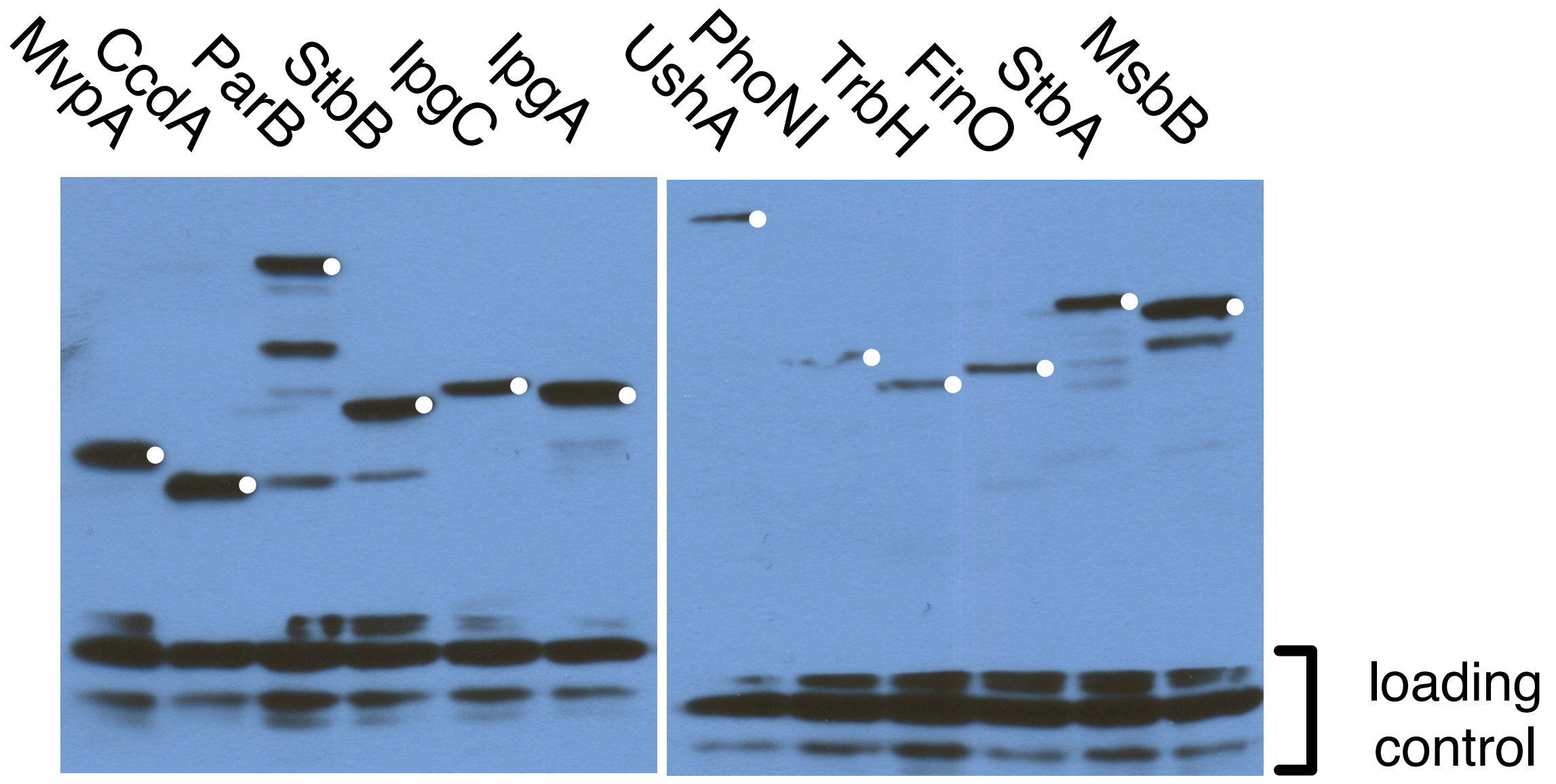

translocated *Shigella* proteins

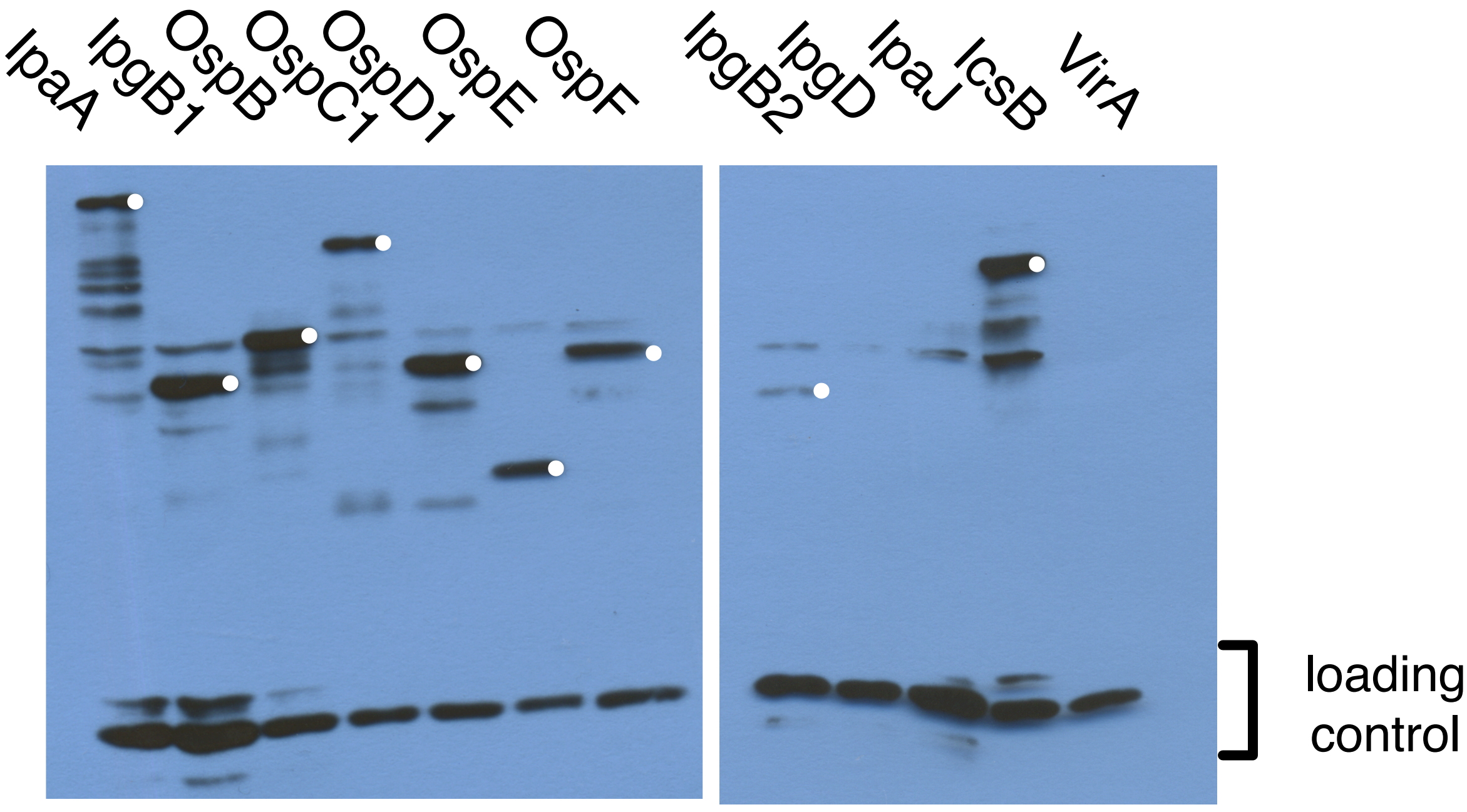

Francisella proteins

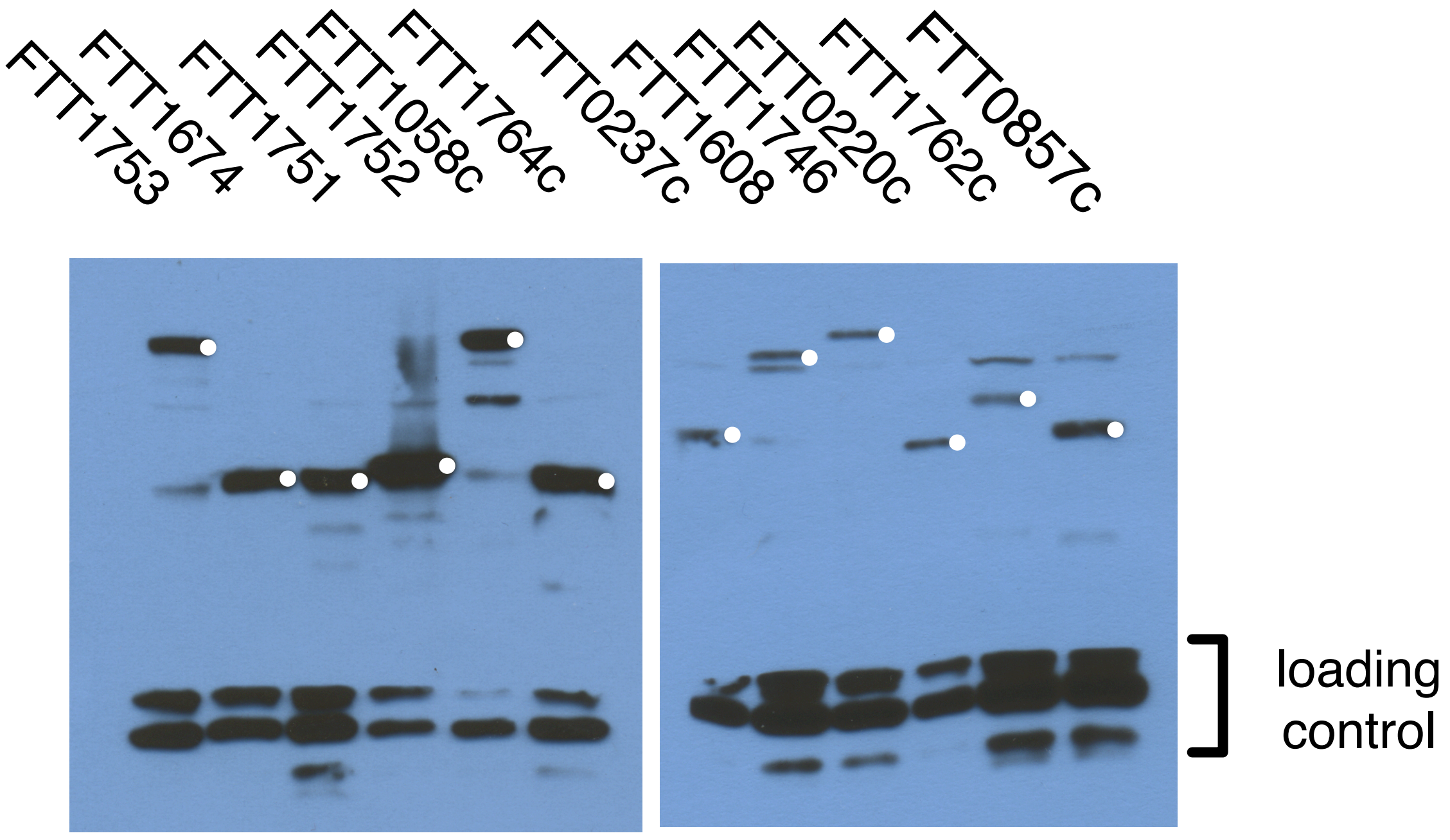

Supplement: Supplementary file 5 [file ppat.0040009.sg005.pdf]
