# Supplementary material for: A Functional Genomic Yeast Screen to Identify Pathogenic Bacterial Proteins
Source: PLoS Pathog. 2008 Jan 18;4(1):e9. doi: 10.1371/journal.ppat.0040009 (PMC2211553; doi:10.1371/journal.ppat.0040009)
Supplement: Supplementary file 6 [file ppat.0040009.st002.doc]

**Table 2.** Summary of Functions of Non-translocated Proteins

| Protein | Function |
| --- | --- |
| acp | Periplasmic acid phosphatase |
| ccdA | Antitoxin-plasmid segregation |
| fin0 | Transfer of plasmids |
| ipgA | TTSS chaperone |
| ipgC | TTSS chaperone |
| ipgE | TTSS chaperone |
| ipgF | TTSS chaperone |
| msbB | Lipid A modification |
| mvpA | Antitoxin-plasmid segregation |
| mvpT | Toxin- plasmid segregation |
| parA | Plasmid segregation |
| parB | Plasmid segregation |
| PhoN2 | Periplasmic acid phosphatase |
| stbA | Plasmid segregation |
| stbB | Plasmid segregation |
| traX | Transfer of plasmids |
| trbH | Unknown |
| ushA | Periplasmic UDP-sugar hydrolase |
| virB | Transcriptional activator |
| virF | Transcriptional activator |

doi:10.1371/journal.ppat.0040009.t002
